# 1,2:1′,2′-Di-*O*-iso­propyl­idenedi­furan­ose-C12 higher carbon sugar

**DOI:** 10.1107/S1600536813021776

**Published:** 2013-08-10

**Authors:** Qiurong Zhang, Guangqiang Zhou, Peng He, Xuebin Chen, Hongmin Liu

**Affiliations:** a New Drug Reseach & Development Center, Zhengzhou Univresity, Zhengzhou 450001, People’s Republic of China

## Abstract

In the title compound, C_18_H_28_O_8_, the five-membered ring with one O atom attached to the ethyl substituent has a twisted conformation about the C—O bond. The adjacent *cis*-fused ring with two O atoms also has a twisted conformation about one of the C—O bonds. The dihedral angle between these rings (all atoms) is 59.05 (12)°. The five-membered ring linked to the ethynyl susbtituent is twisted about a C—C bond; the *cis*-fused adjacent ring is twisted about a C—O bond [dihedral angle between the rings (all atoms) = 71.78 (12)°]. Two intra­molecular O—H⋯O hydrogen bonds occur. In the crystal, mol­ecules are linked by O—H⋯O hydrogen bonds, generating [001] chains.

## Related literature

For further synthetic details, see: Meyer & Jochims (1969 ▶). For background to higher-carbon sugars, see: Iwasa *et al.* (1978 ▶); Harada *et al.* (1981 ▶); Liu *et al.* (2006 ▶).
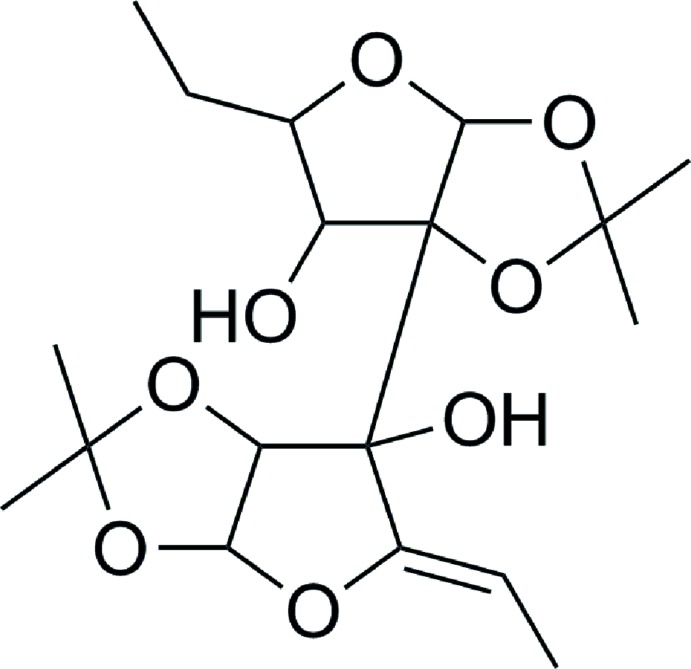


## Experimental

### 

#### Crystal data

C_18_H_28_O_8_
*M* *_r_* = 372.40
Orthorhombic, 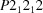

*a* = 21.5802 (6) Å
*b* = 15.3758 (4) Å
*c* = 5.73626 (14) Å
*V* = 1903.37 (8) Å^3^
*Z* = 4
Cu *K*α radiation
μ = 0.86 mm^−1^
*T* = 291 K
0.28 × 0.25 × 0.25 mm

#### Data collection

Agilent Xcalibur (Eos, Gemini) diffractometer
Absorption correction: multi-scan (*SADABS*; Sheldrick, 1996) ▶ *T*_min_ = 0.796, *T*_max_ = 0.815
7406 measured reflections
3407 independent reflections
3079 reflections with *I* > 2σ(*I*)
*R*_int_ = 0.026

#### Refinement

*R*[*F* ^2^ > 2σ(*F* ^2^)] = 0.037
*wR*(*F* ^2^) = 0.099
*S* = 1.05
3407 reflections
262 parameters
H atoms treated by a mixture of independent and constrained refinement
Δρ_max_ = 0.18 e Å^−3^
Δρ_min_ = −0.14 e Å^−3^

### 

Data collection: *CrysAlis PRO* (Agilent, 2011 ▶); cell refinement: *CrysAlis PRO*; data reduction: *CrysAlis PRO*; program(s) used to solve structure: *SHELXS97* (Sheldrick, 2008 ▶); program(s) used to refine structure: *SHELXL97* (Sheldrick, 2008 ▶); molecular graphics: *OLEX2* (Dolomanov *et al.*, 2009 ▶); software used to prepare material for publication: *OLEX2*.

## Supplementary Material

Crystal structure: contains datablock(s) I, global. DOI: 10.1107/S1600536813021776/hb7100sup1.cif

Structure factors: contains datablock(s) I. DOI: 10.1107/S1600536813021776/hb7100Isup2.hkl

Supplementary material file. DOI: 10.1107/S1600536813021776/hb7100Isup3.cml

Additional supplementary materials:  crystallographic information; 3D view; checkCIF report

## Figures and Tables

**Table 1:** Hydrogen-bond geometry (Å, °)

*D*—H⋯*A*	*D*—H	H⋯*A*	*D*⋯*A*	*D*—H⋯*A*
O3′—H3′⋯O4′^i^	0.79 (3)	2.36 (3)	3.038 (2)	144 (2)
O3′—H3′⋯O2′	0.79 (3)	2.22 (3)	2.685 (2)	119 (2)
O3—H3⋯O2	0.82 (4)	2.11 (3)	2.647 (2)	122 (3)

Symmetry code: (i) 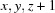.

**Fig. 1.**
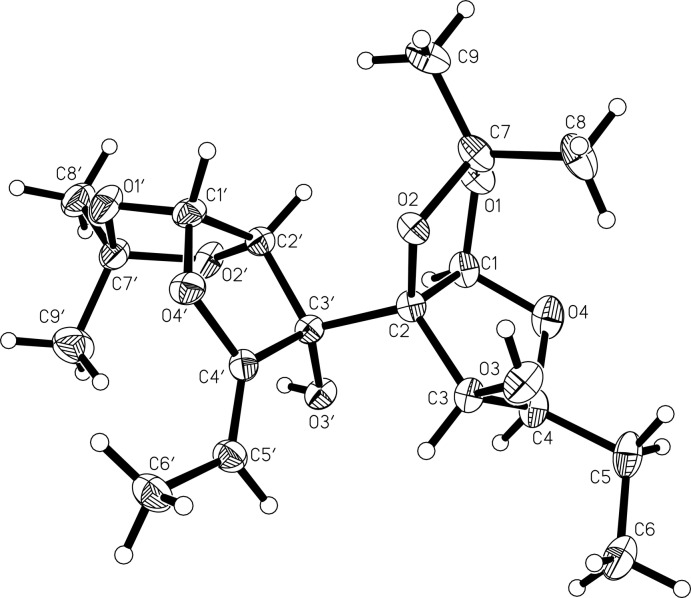
The molecular structure of (I) showing 30% probability displacement ellipsoids.

**Fig. 2.**
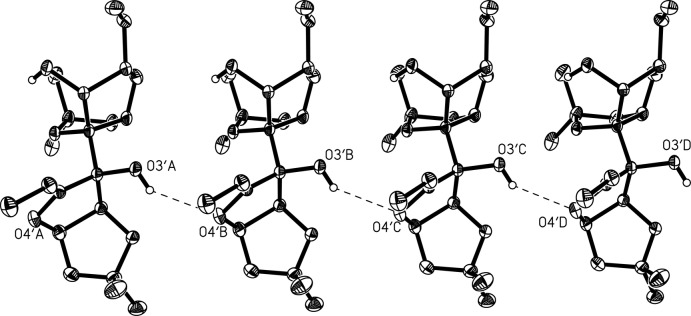
Packing diagram.

## supplementary crystallographic information

### 1. Comment

The term higher carbon sugars is customarily employed with monosaccharides containing seven or more consecutive carbon atoms in the chain. Higher carbon sugars have been attracting the increasing attention of organic chemists in the past decades due to the fact that they can be used as non-metabolized analogues of di- and oligosaccharides and are components of some antibiotics (Iwasa *et al.*, 1978; Harada *et al.*, 1981) and also that they are carbohydrate precursors for higher carbon amino sugars (Liu *et al.*, 2006).

C_18_H_28_O_8_,the title compound (I), is a free C12 higher carbon sugar,whose structure consists of a fused system made up of two methylenedioxy ring and two tetrahydrofuran rings. Both of them, one methylenedioxy ring connects parallelly to tetrahydrofuran, give two fragments with V-shaped models. In the crystal, O—H···O hydrogen bonds (Table 1), link the molecules into [001] chains.

The crystal packing is shown in Figure 2.

### 2. Experimental

The title compound (I) was synthesized from 5,6;5',6'-di-alkene-C12 higher carbon sugar as described previously (Meyer & Jochims, 1969), whose starting material was D-Glucose. A solution of 5,6;5',6'-di-alkene-C12 higher carbon sugar(400 mg, 1 mmol) in aq. CH_3_OH(10 ml) was stirred at room temperature overnight

A suspension of 5,6;5',6'-di-alkene-C12 higher carbon sugar(400 mg, 1 mmol) and NaBH_4_ (0.080 g, 2.08 mmol) in anhydrous MeOH (15 ml) was stirred at room temperature for 1 h. the solvent was evaporated and water (10 ml) was added to the residue, and the mixture was extracted with EtOAc. The combined organic layers were washed with water and dried with anhydrous Na_2_SO_4_. After filtration and evaporation of the solvent, the residue

was dissolved in methanol (20 ml) and 5% Pd/C [500 mg, suspended in methanol (5 ml)] was added. The mixture was degassed, and stirred under an atmosphere of hydrogen. After 4 h, the mixture was filtered and evaporated, Purification of the residue by column chromatography gave the title compound as white solid. Colourless prisms were grown by slow evaporation from CH_3_OH solution at room temperature for two weeks. mp: 398 K; *R*_f_ = 0.40 (petroleum ether/EtOAc, 2:1); ^1^H NMR (400 MHz, CDCl_3_)σ: 5.98 (1*H*, d, *J* = 3.6 Hz), 5.78 (1*H*, s), 4.84 (1*H*, q, *J* = 6.9 Hz), 4.54 (1*H*, d, *J* = 3.6 Hz), 4.22 (1*H*, t), 3.74(1*H*, m), 2.89 (1*H*, s), 2.22 (1*H*, d, *J* = 3.2 Hz), 1.78 2H, m), 1.70 (3*H*, d), 1.68 (3*H*, s), 1.58 (3*H*, s), 1.48 (3*H*, s), 1.46 (3*H*, s), 1.03 (3*H*, d, *J* = 7.5 Hz); ^13^C NMR (100 MHz, CDCl_3_) σ: 154.51, 117.02, 114.03, 106.48, 104.54, 98.57, 94.88, 83.93, 80.18, 79.70, 70.21, 28.03, 27.97, 27.82, 27.62, 21.85, 10.33, 10.23.

### 3. Refinement

All H atoms were placed geometrically and treated as riding on their parent atoms with C—H are 0.96 Å (methylene) or 0.93 Å (aromatic), 0.82 Å (hydroxyl)and *U*_iso_(H) =1.2*U*_eq_(C). Attempts to confirm the absolute structure by refinement of the Flack parameter in the presence of 1412 sets of Friedel equivalents led to an inconclusive value of 0.3 (2). Therefore, the absolute configuration was assigned to correspond with that of the known chiral centres in a precursor molecule, which remained unchanged during the synthesis of the title compound.

### Crystal data

**Table:**

C_18_H_28_O_8_	*D*_x_ = 1.300 Mg m^−^^3^
*M**_r_* = 372.40	Cu *K*α radiation, λ = 1.54184 Å
Orthorhombic, *P*2_1_2_1_2	Cell parameters from 3059 reflections
*a* = 21.5802 (6) Å	θ = 2.9–66.9°
*b* = 15.3758 (4) Å	µ = 0.86 mm^−^^1^
*c* = 5.73626 (14) Å	*T* = 291 K
*V* = 1903.37 (8) Å^3^	Prism, colourless
*Z* = 4	0.28 × 0.25 × 0.25 mm
*F*(000) = 800	

### Data collection

**Table:**

Agilent Xcalibur (Eos, Gemini) diffractometer	3407 independent reflections
Radiation source: fine-focus sealed tube	3079 reflections with *I* > 2σ(*I*)
Graphite monochromator	*R*_int_ = 0.026
Detector resolution: 0 pixels mm^-1^	θ_max_ = 67.1°, θ_min_ = 3.5°
ω scans	*h* = −24→25
Absorption correction: multi-scan (*SADABS*; Sheldrick, 1996)	*k* = −18→17
*T*_min_ = 0.796, *T*_max_ = 0.815	*l* = −4→6
7406 measured reflections	

### Refinement

**Table:**

Refinement on *F*^2^	Secondary atom site location: difference Fourier map
Least-squares matrix: full	Hydrogen site location: inferred from neighbouring sites
*R*[*F*^2^ > 2σ(*F*^2^)] = 0.037	H atoms treated by a mixture of independent and constrained refinement
*wR*(*F*^2^) = 0.099	*w* = 1/[σ^2^(*F*_o_^2^) + (0.0493*P*)^2^ + 0.1435*P*] where *P* = (*F*_o_^2^ + 2*F*_c_^2^)/3
*S* = 1.05	(Δ/σ)_max_ < 0.001
3407 reflections	Δρ_max_ = 0.18 e Å^−^^3^
262 parameters	Δρ_min_ = −0.14 e Å^−^^3^
0 restraints	Extinction correction: *SHELXL97* (Sheldrick, 2008), Fc^*^=kFc[1+0.001xFc^2^λ^3^/sin(2θ)]^-1/4^
Primary atom site location: structure-invariant direct methods	Extinction coefficient: 0.0064 (4)

### Special details

**Table:**

Geometry. All e.s.d.'s (except the e.s.d. in the dihedral angle between two l.s. planes) are estimated using the full covariance matrix. The cell e.s.d.'s are taken into account individually in the estimation of e.s.d.'s in distances, angles and torsion angles; correlations between e.s.d.'s in cell parameters are only used when they are defined by crystal symmetry. An approximate (isotropic) treatment of cell e.s.d.'s is used for estimating e.s.d.'s involving l.s. planes.
Refinement. Refinement of *F*^2^ against ALL reflections. The weighted *R*-factor *wR* and goodness of fit *S* are based on *F*^2^, conventional *R*-factors *R* are based on *F*, with *F* set to zero for negative *F*^2^. The threshold expression of *F*^2^ > σ(*F*^2^) is used only for calculating *R*-factors(gt) *etc*. and is not relevant to the choice of reflections for refinement. *R*-factors based on *F*^2^ are statistically about twice as large as those based on *F*, and *R*- factors based on ALL data will be even larger.

### Fractional atomic coordinates and isotropic or equivalent isotropic displacement parameters (Å^2^)

**Table:**

	*x*	*y*	*z*	*U*_iso_*/*U*_eq_	
O1	0.67974 (7)	0.17289 (11)	0.8701 (3)	0.0581 (4)	
O1'	0.43970 (8)	0.11163 (12)	0.6552 (3)	0.0671 (5)	
O2	0.63054 (6)	0.23737 (9)	0.5648 (2)	0.0418 (3)	
O2'	0.48323 (7)	0.15439 (10)	0.9957 (3)	0.0589 (4)	
O3	0.66664 (9)	0.40182 (11)	0.5529 (3)	0.0587 (4)	
O3'	0.53146 (7)	0.31495 (9)	1.0267 (2)	0.0484 (3)	
O4	0.69581 (7)	0.31202 (12)	1.0229 (3)	0.0659 (5)	
O4'	0.48425 (7)	0.23484 (9)	0.4771 (2)	0.0520 (3)	
C1	0.65181 (9)	0.24742 (14)	0.9636 (4)	0.0496 (5)	
H1	0.6260	0.2325	1.0984	0.060*	
C1'	0.49427 (10)	0.15364 (13)	0.5914 (4)	0.0506 (5)	
H1'	0.5199	0.1155	0.4944	0.061*	
C2	0.61210 (9)	0.28500 (12)	0.7651 (3)	0.0402 (4)	
C2'	0.52685 (9)	0.17335 (13)	0.8211 (3)	0.0436 (4)	
H2'	0.5644	0.1382	0.8392	0.052*	
C3	0.63097 (9)	0.38264 (13)	0.7514 (3)	0.0440 (4)	
H3A	0.5938	0.4192	0.7538	0.053*	
C3'	0.54226 (9)	0.27154 (13)	0.8134 (3)	0.0398 (4)	
C4	0.66772 (10)	0.39550 (15)	0.9764 (4)	0.0522 (5)	
H4	0.6386	0.4087	1.1025	0.063*	
C4'	0.49988 (9)	0.30348 (13)	0.6225 (3)	0.0430 (4)	
C5	0.71713 (12)	0.4649 (2)	0.9732 (5)	0.0689 (7)	
C5'	0.47675 (10)	0.38174 (15)	0.5919 (4)	0.0528 (5)	
C6	0.69055 (13)	0.55472 (19)	0.9364 (6)	0.0798 (8)	
H6A	0.6581	0.5649	1.0476	0.120*	
H6B	0.6741	0.5591	0.7814	0.120*	
H6C	0.7226	0.5973	0.9570	0.120*	
C6'	0.43255 (12)	0.40709 (18)	0.4031 (5)	0.0679 (7)	
H6'A	0.4021	0.4463	0.4648	0.102*	
H6'B	0.4124	0.3560	0.3437	0.102*	
H6'C	0.4548	0.4352	0.2794	0.102*	
C7	0.68335 (10)	0.18190 (16)	0.6219 (4)	0.0544 (5)	
C7'	0.42493 (10)	0.13080 (15)	0.8936 (4)	0.0561 (6)	
C8	0.74382 (10)	0.2237 (2)	0.5493 (5)	0.0704 (7)	
H8A	0.7776	0.1860	0.5890	0.106*	
H8B	0.7486	0.2782	0.6288	0.106*	
H8C	0.7436	0.2335	0.3840	0.106*	
C8'	0.40403 (12)	0.04898 (16)	1.0145 (6)	0.0737 (8)	
H8'A	0.4364	0.0063	1.0071	0.111*	
H8'B	0.3676	0.0268	0.9390	0.111*	
H8'C	0.3948	0.0617	1.1746	0.111*	
C9	0.67296 (12)	0.09468 (15)	0.5109 (5)	0.0677 (7)	
H9A	0.6340	0.0715	0.5621	0.101*	
H9B	0.7057	0.0558	0.5552	0.101*	
H9C	0.6725	0.1010	0.3444	0.101*	
C9'	0.37906 (14)	0.2033 (2)	0.9154 (7)	0.0895 (10)	
H9'A	0.3409	0.1868	0.8420	0.134*	
H9'B	0.3952	0.2545	0.8408	0.134*	
H9'C	0.3717	0.2153	1.0772	0.134*	
H5'	0.4875 (10)	0.4262 (15)	0.710 (4)	0.053 (6)*	
H3'	0.5069 (12)	0.2890 (17)	1.101 (5)	0.058 (7)*	
H3	0.6667 (15)	0.360 (2)	0.465 (7)	0.098 (11)*	
H5A	0.7420 (12)	0.4587 (17)	1.114 (5)	0.062 (7)*	
H5B	0.7476 (13)	0.4461 (19)	0.861 (6)	0.073 (9)*	

### Atomic displacement parameters (Å^2^)

**Table:**

	*U*^11^	*U*^22^	*U*^33^	*U*^12^	*U*^13^	*U*^23^
O1	0.0578 (8)	0.0739 (10)	0.0424 (8)	0.0150 (7)	−0.0015 (7)	0.0186 (7)
O1'	0.0701 (10)	0.0719 (10)	0.0592 (10)	−0.0228 (8)	−0.0003 (8)	−0.0176 (9)
O2	0.0427 (6)	0.0527 (7)	0.0299 (6)	0.0081 (5)	−0.0010 (5)	0.0049 (6)
O2'	0.0650 (9)	0.0692 (9)	0.0425 (8)	−0.0251 (7)	0.0092 (7)	−0.0090 (7)
O3	0.0817 (11)	0.0624 (9)	0.0319 (8)	−0.0183 (8)	0.0031 (8)	0.0065 (7)
O3'	0.0597 (8)	0.0523 (7)	0.0330 (7)	−0.0070 (7)	0.0067 (7)	−0.0056 (6)
O4	0.0618 (9)	0.0881 (11)	0.0478 (9)	−0.0116 (8)	−0.0254 (8)	0.0167 (9)
O4'	0.0642 (8)	0.0547 (8)	0.0370 (7)	0.0048 (6)	−0.0076 (7)	−0.0093 (6)
C1	0.0485 (10)	0.0668 (12)	0.0334 (10)	−0.0007 (9)	−0.0060 (8)	0.0112 (9)
C1'	0.0598 (11)	0.0494 (10)	0.0426 (11)	−0.0011 (9)	0.0042 (10)	−0.0135 (8)
C2	0.0438 (9)	0.0489 (10)	0.0279 (8)	−0.0001 (8)	−0.0022 (8)	0.0044 (7)
C2'	0.0465 (10)	0.0446 (10)	0.0396 (10)	−0.0009 (8)	0.0041 (8)	−0.0010 (8)
C3	0.0470 (10)	0.0539 (10)	0.0311 (9)	−0.0067 (8)	−0.0039 (8)	0.0044 (9)
C3'	0.0451 (10)	0.0434 (9)	0.0310 (9)	−0.0013 (8)	0.0014 (7)	−0.0023 (8)
C4	0.0516 (11)	0.0718 (13)	0.0334 (10)	−0.0135 (10)	−0.0070 (9)	0.0048 (10)
C4'	0.0397 (9)	0.0521 (11)	0.0371 (9)	−0.0013 (8)	0.0010 (8)	−0.0058 (8)
C5	0.0569 (13)	0.1004 (19)	0.0494 (14)	−0.0283 (13)	−0.0095 (12)	−0.0036 (14)
C5'	0.0528 (11)	0.0533 (11)	0.0525 (12)	0.0079 (9)	−0.0060 (10)	−0.0030 (10)
C6	0.0788 (16)	0.0816 (18)	0.079 (2)	−0.0332 (14)	0.0106 (16)	−0.0152 (15)
C6'	0.0632 (13)	0.0748 (16)	0.0657 (16)	0.0218 (12)	−0.0114 (12)	0.0000 (13)
C7	0.0501 (11)	0.0722 (14)	0.0408 (11)	0.0182 (10)	0.0019 (9)	0.0149 (10)
C7'	0.0547 (11)	0.0557 (12)	0.0579 (13)	−0.0105 (10)	0.0084 (11)	−0.0128 (11)
C8	0.0471 (11)	0.1052 (19)	0.0590 (15)	0.0145 (12)	0.0032 (11)	0.0242 (15)
C8'	0.0734 (15)	0.0600 (13)	0.088 (2)	−0.0209 (11)	0.0128 (15)	−0.0109 (14)
C9	0.0784 (15)	0.0659 (14)	0.0587 (15)	0.0272 (12)	0.0125 (13)	0.0090 (12)
C9'	0.0757 (17)	0.0783 (18)	0.114 (3)	0.0070 (14)	0.0291 (19)	0.0034 (18)

### Geometric parameters (Å, º)

**Table:**

O1—C1	1.401 (3)	C4'—C5'	1.315 (3)
O1—C7	1.433 (3)	C5—C6	1.510 (4)
O1'—C1'	1.392 (3)	C5—H5A	0.98 (3)
O1'—C7'	1.434 (3)	C5—H5B	0.96 (3)
O2—C2	1.420 (2)	C5'—C6'	1.495 (3)
O2—C7	1.461 (2)	C5'—H5'	0.99 (2)
O2'—C2'	1.405 (2)	C6—H6A	0.9600
O2'—C7'	1.435 (3)	C6—H6B	0.9600
O3—C3	1.406 (2)	C6—H6C	0.9600
O3—H3	0.82 (4)	C6'—H6'A	0.9600
O3'—C3'	1.413 (2)	C6'—H6'B	0.9600
O3'—H3'	0.79 (3)	C6'—H6'C	0.9600
O4—C1	1.416 (3)	C7—C9	1.501 (4)
O4—C4	1.444 (3)	C7—C8	1.513 (3)
O4'—C4'	1.387 (2)	C7'—C9'	1.496 (4)
O4'—C1'	1.427 (3)	C7'—C8'	1.506 (4)
C1—C2	1.538 (3)	C8—H8A	0.9600
C1—H1	0.9800	C8—H8B	0.9600
C1'—C2'	1.524 (3)	C8—H8C	0.9600
C1'—H1'	0.9800	C8'—H8'A	0.9600
C2—C3'	1.546 (3)	C8'—H8'B	0.9600
C2—C3	1.557 (3)	C8'—H8'C	0.9600
C2'—C3'	1.547 (3)	C9—H9A	0.9600
C2'—H2'	0.9800	C9—H9B	0.9600
C3—C4	1.528 (3)	C9—H9C	0.9600
C3—H3A	0.9800	C9'—H9'A	0.9600
C3'—C4'	1.509 (3)	C9'—H9'B	0.9600
C4—C5	1.508 (3)	C9'—H9'C	0.9600
C4—H4	0.9800		
			
C1—O1—C7	108.93 (17)	C6—C5—H5A	114.4 (16)
C1'—O1'—C7'	110.09 (16)	C4—C5—H5B	106.1 (18)
C2—O2—C7	109.79 (14)	C6—C5—H5B	116.1 (19)
C2'—O2'—C7'	110.41 (16)	H5A—C5—H5B	99 (2)
C3—O3—H3	109 (2)	C4'—C5'—C6'	125.3 (2)
C3'—O3'—H3'	109.9 (19)	C4'—C5'—H5'	116.9 (14)
C1—O4—C4	107.33 (15)	C6'—C5'—H5'	117.7 (14)
C4'—O4'—C1'	110.65 (15)	C5—C6—H6A	109.5
O1—C1—O4	112.17 (17)	C5—C6—H6B	109.5
O1—C1—C2	105.30 (17)	H6A—C6—H6B	109.5
O4—C1—C2	106.74 (16)	C5—C6—H6C	109.5
O1—C1—H1	110.8	H6A—C6—H6C	109.5
O4—C1—H1	110.8	H6B—C6—H6C	109.5
C2—C1—H1	110.8	C5'—C6'—H6'A	109.5
O1'—C1'—O4'	113.50 (18)	C5'—C6'—H6'B	109.5
O1'—C1'—C2'	104.77 (17)	H6'A—C6'—H6'B	109.5
O4'—C1'—C2'	107.05 (16)	C5'—C6'—H6'C	109.5
O1'—C1'—H1'	110.4	H6'A—C6'—H6'C	109.5
O4'—C1'—H1'	110.4	H6'B—C6'—H6'C	109.5
C2'—C1'—H1'	110.4	O1—C7—O2	103.70 (17)
O2—C2—C1	104.42 (15)	O1—C7—C9	109.1 (2)
O2—C2—C3'	110.43 (15)	O2—C7—C9	108.06 (19)
C1—C2—C3'	111.12 (15)	O1—C7—C8	111.2 (2)
O2—C2—C3	112.52 (15)	O2—C7—C8	111.28 (18)
C1—C2—C3	104.71 (15)	C9—C7—C8	113.0 (2)
C3'—C2—C3	113.14 (16)	O1'—C7'—O2'	104.26 (17)
O2'—C2'—C1'	105.43 (16)	O1'—C7'—C9'	112.3 (3)
O2'—C2'—C3'	111.52 (16)	O2'—C7'—C9'	110.9 (2)
C1'—C2'—C3'	105.59 (16)	O1'—C7'—C8'	109.5 (2)
O2'—C2'—H2'	111.3	O2'—C7'—C8'	106.6 (2)
C1'—C2'—H2'	111.3	C9'—C7'—C8'	112.7 (2)
C3'—C2'—H2'	111.3	C7—C8—H8A	109.5
O3—C3—C4	111.89 (16)	C7—C8—H8B	109.5
O3—C3—C2	112.73 (17)	H8A—C8—H8B	109.5
C4—C3—C2	102.58 (16)	C7—C8—H8C	109.5
O3—C3—H3A	109.8	H8A—C8—H8C	109.5
C4—C3—H3A	109.8	H8B—C8—H8C	109.5
C2—C3—H3A	109.8	C7'—C8'—H8'A	109.5
O3'—C3'—C4'	112.00 (15)	C7'—C8'—H8'B	109.5
O3'—C3'—C2	104.63 (15)	H8'A—C8'—H8'B	109.5
C4'—C3'—C2	114.66 (15)	C7'—C8'—H8'C	109.5
O3'—C3'—C2'	113.63 (16)	H8'A—C8'—H8'C	109.5
C4'—C3'—C2'	102.01 (15)	H8'B—C8'—H8'C	109.5
C2—C3'—C2'	110.20 (15)	C7—C9—H9A	109.5
O4—C4—C5	109.52 (19)	C7—C9—H9B	109.5
O4—C4—C3	104.99 (17)	H9A—C9—H9B	109.5
C5—C4—C3	116.62 (19)	C7—C9—H9C	109.5
O4—C4—H4	108.5	H9A—C9—H9C	109.5
C5—C4—H4	108.5	H9B—C9—H9C	109.5
C3—C4—H4	108.5	C7'—C9'—H9'A	109.5
C5'—C4'—O4'	121.58 (19)	C7'—C9'—H9'B	109.5
C5'—C4'—C3'	128.69 (19)	H9'A—C9'—H9'B	109.5
O4'—C4'—C3'	109.65 (16)	C7'—C9'—H9'C	109.5
C4—C5—C6	112.3 (2)	H9'A—C9'—H9'C	109.5
C4—C5—H5A	108.0 (16)	H9'B—C9'—H9'C	109.5
			
C7—O1—C1—O4	−90.8 (2)	C3—C2—C3'—C2'	179.19 (16)
C7—O1—C1—C2	24.9 (2)	O2'—C2'—C3'—O3'	24.3 (2)
C4—O4—C1—O1	145.01 (18)	C1'—C2'—C3'—O3'	138.29 (16)
C4—O4—C1—C2	30.2 (2)	O2'—C2'—C3'—C4'	−96.47 (18)
C7'—O1'—C1'—O4'	−95.8 (2)	C1'—C2'—C3'—C4'	17.55 (19)
C7'—O1'—C1'—C2'	20.6 (2)	O2'—C2'—C3'—C2	141.33 (16)
C4'—O4'—C1'—O1'	107.62 (19)	C1'—C2'—C3'—C2	−104.65 (18)
C4'—O4'—C1'—C2'	−7.5 (2)	C1—O4—C4—C5	−164.72 (19)
C7—O2—C2—C1	−6.9 (2)	C1—O4—C4—C3	−38.8 (2)
C7—O2—C2—C3'	−126.46 (17)	O3—C3—C4—O4	−90.6 (2)
C7—O2—C2—C3	106.07 (18)	C2—C3—C4—O4	30.5 (2)
O1—C1—C2—O2	−10.73 (19)	O3—C3—C4—C5	30.8 (3)
O4—C1—C2—O2	108.66 (18)	C2—C3—C4—C5	151.9 (2)
O1—C1—C2—C3'	108.34 (18)	C1'—O4'—C4'—C5'	−157.3 (2)
O4—C1—C2—C3'	−132.27 (17)	C1'—O4'—C4'—C3'	19.9 (2)
O1—C1—C2—C3	−129.19 (17)	O3'—C3'—C4'—C5'	32.0 (3)
O4—C1—C2—C3	−9.8 (2)	C2—C3'—C4'—C5'	−87.0 (3)
C7'—O2'—C2'—C1'	−4.3 (2)	C2'—C3'—C4'—C5'	153.9 (2)
C7'—O2'—C2'—C3'	109.84 (19)	O3'—C3'—C4'—O4'	−144.87 (16)
O1'—C1'—C2'—O2'	−9.9 (2)	C2—C3'—C4'—O4'	96.09 (19)
O4'—C1'—C2'—O2'	110.96 (17)	C2'—C3'—C4'—O4'	−23.00 (19)
O1'—C1'—C2'—C3'	−128.03 (17)	O4—C4—C5—C6	−177.3 (2)
O4'—C1'—C2'—C3'	−7.2 (2)	C3—C4—C5—C6	63.7 (3)
O2—C2—C3—O3	−4.8 (2)	O4'—C4'—C5'—C6'	−0.5 (4)
C1—C2—C3—O3	107.97 (18)	C3'—C4'—C5'—C6'	−177.0 (2)
C3'—C2—C3—O3	−130.87 (17)	C1—O1—C7—O2	−28.9 (2)
O2—C2—C3—C4	−125.35 (16)	C1—O1—C7—C9	−143.91 (18)
C1—C2—C3—C4	−12.5 (2)	C1—O1—C7—C8	90.7 (2)
C3'—C2—C3—C4	108.61 (18)	C2—O2—C7—O1	21.6 (2)
O2—C2—C3'—O3'	174.56 (14)	C2—O2—C7—C9	137.29 (18)
C1—C2—C3'—O3'	59.2 (2)	C2—O2—C7—C8	−98.0 (2)
C3—C2—C3'—O3'	−58.30 (19)	C1'—O1'—C7'—O2'	−23.2 (3)
O2—C2—C3'—C4'	−62.3 (2)	C1'—O1'—C7'—C9'	97.0 (2)
C1—C2—C3'—C4'	−177.75 (17)	C1'—O1'—C7'—C8'	−137.0 (2)
C3—C2—C3'—C4'	64.8 (2)	C2'—O2'—C7'—O1'	16.3 (2)
O2—C2—C3'—C2'	52.04 (19)	C2'—O2'—C7'—C9'	−104.8 (2)
C1—C2—C3'—C2'	−63.4 (2)	C2'—O2'—C7'—C8'	132.2 (2)

### Hydrogen-bond geometry (Å, º)

**Table:**

*D*—H···*A*	*D*—H	H···*A*	*D*···*A*	*D*—H···*A*
O3′—H3′···O4′^i^	0.79 (3)	2.36 (3)	3.038 (2)	144 (2)
O3′—H3′···O2′	0.79 (3)	2.22 (3)	2.685 (2)	119 (2)
O3—H3···O2	0.82 (4)	2.11 (3)	2.647 (2)	122 (3)

Symmetry code: (i) *x*, *y*, *z*+1.
